# Non-adherence to epileptic medications among adult patients in south west Shewa zone hospitals of central Ethiopia

**DOI:** 10.1038/s41598-025-00558-6

**Published:** 2025-05-19

**Authors:** Nigus Yehenew, Masrie Getnet, Tamiru Tesfaye, Awoke Masrie, Enyew Mekonnen, Desalegn Tamiru, Hailu Merga

**Affiliations:** 1 Department of Public Health, School of Health Sciences, Ambo University Waliso Campus, Waliso, Ethiopia; 2 Department of Epidemiology, Institute of Health, Jimma University, P.O. Box 378, Jimma, Ethiopia; 3 School of Public Health, College of Health and Medical Sciences, Haramaya University, Harar, Ethiopia; 4 Department of Midwifery, Saint Peter’s Specialized Hospital, Federal Minster of Health, Addis Ababa, Ethiopia; 5 Department of Nursing, School of Health Sciences, Ambo University Waliso Campus, P.O. Box 217, Waliso, Ethiopia

**Keywords:** Anti-epileptic, Non-adherence, Epilepsy, Hospitals, South west Shewa, Ethiopia, Epidemiology, Medical research, Risk factors

## Abstract

Epilepsy, a global public health problem, affecting about 50 million humans. Non-adherence to antiepileptic medications leads to treatment failure, hospitalization, decreased productivity and death. Previously conducted studies in Ethiopia did not fully identify these factors, which highlights the need for further research. Hence**,** this study aimed to assess non-adherence to anti-epileptic medications and associated factors among adult patients with epilepsy at the specified study area. Hospital based cross-sectional study was employed to collect data from 361 patients with epilepsy from November 1, 2023 to January 9, 2024. Data was collected using a pretested structured interviewer-administered questionnaire. Systematic random sampling technique was carried out to select study participants. Data was entered into EpiData and exported to Statistical Package for Social Sciences (SPSS) version 25 for analysis. The multivariable logistic regressions were utilized and Adjusted Odds Ratio (AOR), 95% Confidence level and *p*-value < 0.05 was used to declare statistical significance. In this study, 43.1% (95% CI 37.8, 48.4%) of respondents were non-adherent to anti-epileptic medications. Being on treatment for 1–3 years (AOR = 4.1, 95% CI 1.768, 9.427), unable to get health information about their illness (AOR = 2.36, 95% CI 1.4, 3.9) and distance to the health facility (AOR = 2.7, 95% CI 1.56, 4.66) were factors significantly associated with non-adherence to anti-epileptic medications. The prevalence of non-adherence to anti-epileptic medications was found to be high. Duration on treatment, distance to the health facility and unable to get health information about their illness were predictors of non-adherence. Therefore, it is recommended that providing health education and counseling as well solving distance related problems to the health facility is needed to increase adherence for better treatment outcome.

## Introduction

Epilepsy is a chronic, non-communicable brain illness characterized by recurring epileptic seizures caused by an abnormally abrupt and excessive discharge of cerebral neurons or brain cells. It is one of the most common serious neurological disease affecting humans of all ages around the world^1–3^.

Epilepsy affects approximately 50 million people worldwide, nearly 80% of whom live in low and middle-income countries. More than five million new cases are diagnosed each year and the number is expected to continue to rise^2,4^. The prevalence of epilepsy in Sub-Saharan Africa is 15/1000 of the population^5,6^. In Ethiopia, epilepsy is a public health problem, with an estimated prevalence in the country reported to be 5.2/1000 of the population at risk with an annual incidence of 64 per 100,000 population^7^.

According to International League Against Epilepsy, epilepsy is classified as focal, generalized, combined focal and generalized, and unknown^8^. This classification of epilepsy is significant to identify a specific medication that is effective against a specific type of seizure and also helps to identify comorbidities and prognosis with a particular seizure type^9^. The goals of epilepsy treatment are to prevent seizures or minimize their frequency, avoid side effects of long-term therapy, support patients in continuing or resuming their regular psychosocial and occupational activities, and preserve a normal way of life^10,11^. Appropriate antiepileptic drug (AED) therapy is one of the most important and critical components in the management of epilepsy^12^. Patients passive disregard for recommended treatment regimens is reflected in nonadherence to antiepileptic drugs^13^.

Non-adherence to anti-epileptic medications is one of the most serious obstacles in clinical practice and this phenomenon can lead to loss of seizure control in epileptic patients, potentially failing to achieve treatment goals which can otherwise be expected up to 70% of patients receiving optimal AED treatment^14–16^. Failure to comply with AEDs can lead to seizures months or years after the previous episode and can have serious consequences on an individual’s quality of life^17^. The effectiveness of anti-epileptic medications is limited if the patient does not adhere to the treatment regimen^18^.

Despite great concern and willingness to tackle non-adherence, the prevalence of anti-epileptic medications continued to be high. The majority of previously conducted studies on non-adherence to anti-epileptic medications have focused on a single hospital and some of them on a smaller sample size. Furthermore, some of the studies utilized non-probability sampling technique, which can affect the representativeness and generalizability of their finding. Additionally, there is an evidence gap on magnitude and risk factors of non-adherence in the study area specifically.

Therefore, the study aimed to assess the prevalence of non-adherence to anti-epileptic medications and associated factors among adult patients with epilepsy in southwest shewa zone hospitals at multi-center level.

## Study methods and materials

### Study design and setting

A multi-center health facility based cross-sectional study was conducted in Southwest Shewa zone, central Ethiopia from November 1, 2023 to January 9, 2024. Southwest shewa zone has a total population of 1,101,129. Southwest shewa zone comprises five government hospitals named Tullu Bollo General Hospital, Waliso General Hospital, Ameya Primary Hospital, Bantu Primary Hospital, and Leman Primary Hospital, and one non-governmental hospital named Saint Luke’s Catholic Hospital^19^. There were about 1344 epileptic patients on epileptic follow-up in south west Shewa zone hospitals.

### Study participants and sample size

The source population for this study was all patients with epilepsy who were attending hospitals in southwest shewa zone during the study period. All adult patients (18 and above years of age) attending psychiatry/neurology clinic for the management of epilepsy and who were on follow up for at least three months with at least one anti-epileptic medication were eligible for the study. Epileptic patients who were critically ill and could not communicate due to illness were excluded from the study.

The sample size was calculated by EpiInfo version 7.2.5 software with the following assumptions: 44.23% proportion of male`s non-adherence to anti-epileptic medications and 28.75% proportion female’s non-adherence to anti-epileptic medications from the previous study^20^. Furthermore, 95% confidence level and power 80% were used. By considering 10% non-response rate, the sample size became 361.

Systematic random sampling technique was used to recruit each patient with epilepsy at each hospital in southwest shewa zone. The sample size was proportionally allocated based on total number of patients with epilepsy who were on follow up in each six hospitals based on district health information system report. The total number of patients with epilepsy in each hospital were:—210 in Saint Luke’s Catholic hospital, 240 in Waliso General hospital, 220 in Tullu Bollo hospital, 224 in Ameya hospital, 216 in Bantu hospital, and 234 in Leman hospital. Then, the sampling interval was determined by dividing the total number of patients with epilepsy at each hospital by the calculated sample size. Accordingly, every 4 patient was interviewed following clinician’s visit after the first participant was selected by lottery method and special mark was used in the chart to avoid recycling the data.

### Measurement

The dependent variable for this study is non-adherence to anti-epileptic medications. Medication adherence was assessed in this study to extent a patient’s medication taking behavior conforms with the agreed upon recommendations of a health care provider^21^.

Eight-item Morisky Medication Adherence Scale (MMAS) was used to measure self-reported anti-epileptic medication non-adherence with items 1 to 7 with ‘yes’ and ‘No’ response. A ‘yes’ response was scored 1 and a ‘No’ response was scored 0. Item 8 was measured on a five-point scale and responses “never” was scored 0, but “once in a while”, “sometimes”, “usually”, and “all the time” were scored 1. They were categorized as adhered if MMAS score was 0 and as non-adherent if the MMAS score was 1–8^22^.

### Data collection tool and quality assurance

Interviewer-administered structured questionnaire was prepared according to the objectives of the study adapted from relevant literatures in English language^13,23–29^. Questionnaires were translated to Amharic and Afaan Oromo languages by language experts then translated back to English to ensure consistency. The data was collected by a pre-tested face-to-face interviewer administered structured questionnaire along with patient’s chart review.

The questionnaire has four parts. It includes socio-demographic and economic, clinical and treatment related factors, health care and patient related factors and Morisky eight-item medication adherence questionnaire (MMAS-8). Non-adherence to AEMs was measured by an eight-item Morisky Medication Adherence Scale (MMAS-8)^22,30^.

The data collectors were bachelor degree holder nurses who were fluent in the local language. Additionally, two supervisors with master’s degree in public health were employed for the data collection. The six data collectors and two supervisors were given training on the study objectives, method of data collection, and the tools for data collection. Eligible study participants from the outpatient department were linked to the data collectors and the interview was conducted after patients received routine care in the facility. The data collection tool was pre-tested in 5% of the total sample size in Ambo general hospital (nearby study setting institution); based on these results, adjustments were made to the data collection tool.

### Data processing and analysis

Data was entered into EpiData and then exported to SPSS version 25 for analysis. Binary logistic regression (bivariable and multivariable) analysis was performed to determine association between the independent variables and the dependent variable. All variables associated with non-adherence at a *P*-value ≤ 0.25 on the bivariable analysis were entered into a multivariable logistic regression analysis to control for confounders. Finally, the predictors of non-adherence to AEDs were declared with Adjusted Odds Ratio (AOR), 95% Confidence interval and *p*-value < 0.05. Wealth Index was analyzed using Principal Component Analysis (PCA). The model fitness was checked using Hosmer–Lemeshow goodness of fit test and was found fit (0.256). A multicollinearity test was done using variance inflation factor (VIF) and its value was less than ten (1.89).

## Results

### Socio-demographic and socio-economic characteristics of respondents

Among 361 sample of patients with epilepsy, 353 respondents were participated in this study with a response rate of 97.7%. The mean (SD) age of the respondents was 30.8 (SD ± 11.19) year, ranging from 18 to 72 years. More than half 212 (60.1%) of the respondents were males and 158 (44.8%) were single. About one third of respondents were farmers 129 (36.5%) and more than half 205 (58.1%) were rural residents (Table 1).

**Table 1:** Socio-demographic and socio-economic characteristics of respondents at hospitals of southwest Shewa zone, Oromia, central Ethiopia, 2024 (N = 353).

Variables	Category	Frequency (%)
Age	18–22	93 (26.3)
	23–27	66 (18.7)
	28–32	66 (18.7)
	33–37	48 (13.7)
	38–42	28 (7.9)
	≥ 43	52 (14.7)
	Mean ± SD	30.80 ± 11.19
Sex	Male	212 (60.1)
	Female	141 (39.9)
Marital status	single	158 (44.8)
	Married	174 (49.3)
	Divorced	14 (4.0)
	Widowed	7 (2.0)
Religion	Orthodox	157 (44.5)
	Protestant	144 (40.8)
	Muslim	46 (13.0)
	Wakefeta	6 (1.7)
Ethnicity	Oromo	298 (84.4)
	Amhara	35 (9.9)
	Gurage	20 (5.7)
Occupation	Government employee	12 (3.4)
	Private business	65 (18.4)
	Farmer	129 (36.5)
	Daily laborer	21 (5.9)
	Student	81 (22.9)
	Household worker	31 (8.9)
	Jobless	14 (4.0)
Wealth index	Lowest	64 (18.1)
	Second	80 (22.7)
	Middle	68 (19.2)
	Fourth	74 (21.0)
	Highest	67 (19.0)
Educational level	Unable to read and write	88 (24.9)
	Grade 1–8	171 (48.4)
	Grade 9–12	55 (15.7)
	College and above	39 (11.0)
Place of residence	Rural	205 (58.1)
	Urban	148 (41.9)

### Non-adherence to anti-epileptic medications and clinical characteristics of respondents

The prevalence of non-adherence to anti-epileptic medications in this study was 43.1%, 95% CI 37.8, 48.4%). More than two-thirds of participants, 71.4% were on monotherapy. Majority of the respondents, 89.2% did not take medications other than anti-epileptic drugs. About 19.5% of the study subjects reported side effects of AEMs like dizziness, confusion, weakness, blurred vision, insomnia, headache, forgetfulness and excessive sleep (Table 2).

**Table 2:** Clinical and treatment related characteristics of respondents at hospitals of southwest Shewa zone, Oromia, central Ethiopia, 2024 (N = 353).

Variables	Category	Frequency (%)
Current anti-epileptic drugs	Phenobarbitone	151 (42.8)
	Phenytoin	62 (17.6)
	Sodium valproate	8 (2.3)
	Carbamazepine	30 (8.5)
	Phenobarbitone + phenytoin	53 (15.0)
	Phenobarbitone + sodium valproate	17 (4.8)
	Phenobarbitone + carbamazepine	26 (7.4)
	Phenytoin + sodium valproate	3 (0.8)
	Phenytoin + carbamazepine	3 (0.8)
Medication other than anti-epileptic drugs	No	315 (89.2)
	Yes	38 (10.8)
Type of medications other than anti-epileptic drugs	Risperidone	9 (2.5)
	Haloperidol	5 (1.4)
	Olanzapine	1 (0.3)
	Amitriptyline	8 (2.3)
	Chlorpromazine	4 (1.1)
	Nifedipine	3 (0.8)
	Amlodipine	6 (1.8)
	Fluoxetine	2 (0.6)
Number of anti-epileptic drugs prescribed	One	252 (71.4)
	Two	101 (28.6)
Comorbid illness	No	315 (89.2)
	Yes	38 (10.8)
Reported side effects	No	284 (80.5)
	Yes	69 (19.5)
Duration on treatment	3 months–1 year	46 (13.0)
	1–3 years	109 (30.9)
	3–5 years	114 (32.3)
	> 5 years	84 (23.8)

### Health care and patient related characteristics of respondents

Among the total respondents, 249 (70.5%) of them were getting medication by payment. Nearly half 151 (42.8%) of the participants did not get health information about their illness. In relation to substance, 59 (16.7%) of them had current substance use history and alcohol 47 (13.3%) was the most commonly used substance. About 151 (42.8%) of the respondents experienced epilepsy-related perceived stigma and 159 (45.1%) of them had poor social support. About 155 (43.9%) of the study subjects reported that distance to the health facility was a big problem (Table 3).

**Table 3:** Health care and patient related characteristics of respondents at hospitals of south west Shewa zone, Oromia, central Ethiopia, 2024 (N = 353).

Variables	Category	Frequency (%)
Getting medication	Freely	104 (29.5)
	By payment	249 (70.5)
Getting health information	Yes	202 (57.2)
	No	151 (42.8)
Ever substance use	Yes	140 (39.7)
	No	213 (60.3)
Current substance use	Yes	59 (16.7)
	No	294 (83.3)
Frequency of alcohol use	Once or twice	36 (10.2)
	Monthly	2 (0.6)
	Weekly	9 (2.5)
Perceived stigma	Not perceived stigmatized	202 (57.2)
	Perceived stigmatized	151 (42.8)
Social support	Poor social support	159 (45.1)
	Moderate social support	148 (41.9)
	Strong social support	46 (13.0)
Perception on distance to the health facility	It is a big problem	155 (43.9)
	It is not a big problem	198 (56.1)

### Factors associated with non-adherence to anti-epileptic medications

In bivariable logistic regression analysis nine variables: occupational status, wealth index, educational level, place of residence, medication other than AEDs, medication side effects, duration on treatment, getting health information and distance to the health facility were associated with non-adherence to AEMs (Table 4). In multivariable logistic regression only three variables were significantly associated with non-adherence to AEMs. Patients who had been on treatment for 1–3 years were 4 times more likely to be non-adherent to AEMs compared to patients who had been on treatment for 3 months to 1 year (AOR = 4.1.95% CI 1.768, 9.43). Patients who did not get health information about their illness were 2.36 times more likely to be non-adherent to AEMs compared to patients who got health information about their illness (AOR = 2.36, 95% CI 1.423, 3.9). Perception of respondents to distance of the health facility was also significantly associated with non-adherence to AEMs, in which the odds of nonadherence to AEMs were 2.7 times more likely among patients who reported distance to the health facility was a big problem compared to those who reported distance to the health facility was not a big problem (AOR = 2.7, 95% CI 1.57, 4.66) (Table 5).

**Table 4:** Bivariable logistic regression analysis of factors associated with non-adherence to AEMs among adult patients with epilepsy at hospitals of southwest Shewa zone, Oromia, central Ethiopia, 2024.

Independent variables	Category	Non-adherence to AEMs		COR (95% CI)
		Yes (%)	No (%)	
Occupation	Government employed	4 (33.3)	8 (66.7)	**1**
	Private business	29 (44.6)	36 (55.4)	1.611 (0.441–5.888)
	Farmer	54 (41.9)	75 (58.1)	1.440 (0.413–5.027)
	Daily laborer	10 (47.6)	11 (52.4)	1.818 (0.416–7.943)
	Student	31 (38.3)	50 (61.7)	1.240 (0.344–4.465)
	House hold worker	15 (48.4)	16 (51.6)	1.875 (0.466–7.540)
	Jobless	9 (64.3)	5 (35.7)	3.600 (0.710–18.254)*
Wealth index	Lowest	26 (40.6)	38 (59.4)	1.079 (0.536–2.173)
	Second	36 (45.0)	44 (55.0)	1.290 (0.667–2.496)
	Middle	35 (51.5)	33 (48.5)	1.672 (0.844–3.314)*
	Fourth	29 (39.2)	45 (60.8)	1.016 (0.516–2.001)
	Highest	26 (38.8)	41 (61.2)	**1**
Educational level	Unable to read and write	35 (39.8)	53 (60.2)	1.179 (0.540–2.576)
	Grade 1–8	79 (46.2)	92 (53.8)	1.533 (0.746–3.150)*
	Grade 9–12	24 (43.6)	31 (56.4)	1.382 (0.595–3.215)
	College and above	14 (35.9)	25 (64.1)	**1**
Place of residence	Rural	94 (45.9)	111 (54.1)	1.314 (0.855–2.019)*
	Urban	58 (39.2)	90 (60.8)	**1**
Medication other than anti-epileptic drugs	No	129 (41.0)	186 (59.0)	**1**
	Yes	23 (60.5)	15 (39.5)	2.211 (1.111–4.400)*
Reported side effects	No	114 (40.1)	170 (59.9)	**1**
	Yes	38 (55.1)	31 (44.9)	1.828 (1.076–3.107)*
Duration on treatment	3 months- 1 year	13 (28.3)	33 (71.7)	**1**
	1–3 years	64 (58.7)	45 (41.3)	3.610 (1.711–7.616)*
	3–5 years	47 (41.2)	67 (58.8)	1.781 (0.848–3.741)*
	> 5 years	28 (33.3)	56 (66.7)	1.269 (0.578–2.785)
Getting health information about their illness	Yes	67 (33.2)	135 (66.8)	**1**
	No	85 (56.3)	66 (43.7)	2.595 (1.680–4.009)*
Perception on distance to the health facility	It is a big problem	90 (58.1)	65 (41.9)	3.037 (1.960–4.707)*
	It is not a big problem	62 (31.3)	136 (68.7)	**1**

*Shows statistically significant at *p*-value ≤ 0.25.

**Table 5:** Multivariable logistic regression analysis of factors associated with non-adherence to AEMs among adult patients with epilepsy at hospitals of southwest Shewa zone, Oromia, central Ethiopia, 2024.

Variables	Category	Non-adherence to AEMs		COR (95% CI)	AOR (95% CI)	*P*-value
		Yes (%)	No (%)			
Duration on treatment	3 months- 1 year	13 (28.3)	33 (71.7)	1.0	1.0	0.001*
	1–3 years	64 (58.7)	45 (41.3)	3.6 (1.7–7.62)	4.1 (1.77, 9.43)	
	3–5 years	47 (41.2)	67 (58.8)	1.78 (0.848–3.74)	1.894 (0.833, 4.308)	0.128
	> 5 years	28 (33.3)	56 (66.7)	1.3 (0.578–2.785)	1.21 (0.487, 2.994)	0.683
Getting health information about their illness	Yes	67 (33.2)	135 (66.8)	1.0	1.0	0.001*
	No	85 (56.3)	66 (43.7)	2.59 (1.680–4.1)*	2.36 (1.423, 3.8)	
Perception to distance to the health facility	Big problem	90 (58.1)	65 (41.9)	3.037 (1.96–4.71)*	2.7 (1.56, 4.66)	0.000*
	Not a big problem	62 (31.3)	136 (68.7)	1.0	1.0	

*Shows statistically significant at *p*-value < 0.05

## Discussion

This finding showed that the prevalence of non-adherence to anti-epileptic medications was 43.1%, which was in line with the studies conducted in Malaysia (42.8%), India (44.3%), South Africa (45.4%), Northwest Ethiopia (38.5%), southern Ethiopia (38.1%), Southwestern Ethiopia (40.27%) and, Northwestern Ethiopia (37.8%)^13,25–27,31–33^. The possible reason for this similarity might be due to the use of similar standardized tools or criteria for assessing medication adherence across the studies and similarity of study design employed. This consistency suggests that non-adherence to AEMs is a common challenge faced by patients with epilepsy worldwide. However, this study was higher than the studies conducted in Dessie (Northeast Ethiopia (37.5%)) and Jimma (Southwestern Ethiopia) (36.8%)^20,34^. The discrepancy might be due to differences in healthcare accessibility particularly in this study area, problems related to distance to the health facility and challenges in obtaining anti-epileptic medications. Additionally, the reason for higher level of non-adherence in this study might be due to inadequate health education and awareness about epilepsy and anti-epileptic medications. On the other hand, this finding was lower than the studies conducted in Nigeria (67.4%), Kenya (54%) and Yirgalem (Southern Ethiopia) (68%)^23,28,35^. This disparity might be due to changes in healthcare practices or policies over time could contribute to differences in adherence level of studies conducted at different times. Another reason could be variations in the healthcare systems such as availability of medications, quality of care and patient education programs could influence medication adherence level and also might be due to socio-cultural differences.

The study revealed that patients who had been on treatment for 1 to 3 years were four times more likely to be non-adherent to AEMs compared to patients who had been on treatment for 3 months to 1 year. This finding was consistent with the study done in Yirgalem (Southern Ethiopia)^28^. This might be due to patients who had been on treatment for long time may experience medication fatigue or complacency over time. They may feel that their condition is under control and become less diligent about taking their medication, which makes them non-adherent.

The finding of this study showed that respondents who did not get health information about their illness were two and half times more likely to be non-adherent to AEMs compared to patients who got health information about their illness. This result was similar with the findings from Northwest Ethiopia (Gondar, Debremarkos and Fenoteselam) and Southern Ethiopia^13,25,27^. The possible explanation for this might be patients who did not actively seek or receive health information about their illness may had gaps in their understanding of epilepsy and its treatment. Thus, lack of proper counseling or health information about their illness could lead to misconceptions or uncertainty about the benefits of medication adherence, which contributes to non-adherence to AEMs.

Non-adherence to AEMs was also affected by distance to the health facility. The odds of non-adherence to AEMs were three times more likely among respondents who reported distance to the health facility was a big problem compared to those who reported distance to the health facility was not a big problem. This finding was similar with the study done in Kenya^23^. This similarity might be due to patients who reported distance to the health facility was a big problem might face challenges in accessing care and obtaining their medications. They may be more likely to miss appointments or delay refilling their anti-epileptic medications, which in turn contributes to non-adherence to AEMs.

The study findings are possibly limited by the fact that since non-adherence related questions were self-reported, this may had led to recall bias and socially desirable answers. Hence, it might result in overestimation or under estimation of non-adherence to AEMs.

## Conclusion

The finding of this study indicated that the prevalence of non-adherence to anti-epileptic medications was found to be high. Duration on treatment 1–3 years, having perception that distance to the health facility was a big problem and unable to get health information about their illness were factors significantly associated with non-adherence to antiepileptic medications. Hence, a collaborative and more effort are needed to decrease non-adherence to AEMs through health information and counseling for the patients. Improving accessibility is crucial, as medical facilities ought to be easily accessible to patients, particularly those who have faced transportation or distance-related obstacles.

## Data Availability

The dataset(s) supporting the conclusions of this article are included within the article.

## Abbreviations

AED: Antiepileptic drug
AEMS: Anti-epileptic medications
AOR: Adjusted odds ratio
CI: Confidence interval
COR: Crude odds ratio
MMAS: Morisky medication adherence scale
SPSS: Statistical package for social sciences
